# Association of Vitamin D Status with Chronic Disease Risk Factors and Cognitive Dysfunction in 50–70 Year Old Adults

**DOI:** 10.3390/nu11010141

**Published:** 2019-01-11

**Authors:** Japneet Kaur, Steven L. Ferguson, Eduardo Freitas, Ryan Miller, Debra Bemben, Allen Knehans, Michael Bemben

**Affiliations:** 1 Department of Health and Exercise Science, University of Oklahoma, Norman, OK 73019, USA; eduardofreitas@ou.edu (E.F.); ryanmiller1@ou.edu (R.M.); dbemben@ou.edu (D.B.); mgbemben@ou.edu (M.B.); 2 Helen and Arthur E. Johnson Beth-El College of Nursing and Health Sciences, University of Colorado, Colorado Springs, CO 80918, USA; sfergus7@uccs.edu; 3 Department of Nutritional Sciences, University of Oklahoma Health Sciences Center, Oklahoma City, OK 73104, USA; allen-knehans@ouhsc.edu

**Keywords:** physical activity, elderly, 1,25 dihydroxy vitamin D, 25 hydroxy vitamin D

## Abstract

Vitamin D deficiency/insufficiency has been primarily associated with skeletal disorders, however, since vitamin D receptors are found on multiple types of cells, there is also a link to increased chronic disease risk and all-cause mortality. The aim of this study was to examine whether deficient/insufficient vitamin D levels are associated with risk factors of chronic diseases and cognitive dysfunction in 50 to 70 year old adults. Participants completed the health status, three-day dietary record and vitamin D food frequency, sun exposure, and international physical activity questionnaires. Cognitive function of the participants was assessed using the Automated Neuropsychological Assessment Metrics while body composition (percent body fat, android/gynoid ratio) was assessed using Dual Energy X-ray Absorptiometry. Applanation tonometry was used to obtain pressure wave forms at the radial artery to examine arterial stiffness and central pressures. A fasting blood draw was taken to measure vitamin D, blood lipid and glucose levels. Fifty percent of the participants (36/72) were vitamin D deficient/insufficient. Individuals in the low physical activity (PA) group had lower serum vitamin D concentration compared to those in the high PA group (*p* = 0.04). Moreover, serum vitamin D levels were negatively related to risk factors of chronic diseases; blood glucose (*r* = −0.38; *p* = 0.01), triglycerides (*r* = −0.27; *p* = 0.02), and android/gynoid ratio (*r* = −0.32; *p* = 0.01). Deficient/insufficient vitamin D levels are linked to the risk factors of chronic diseases in men and women aged 50 to 70 years.

## 1. Introduction

The National Health and Nutrition Examination Survey 2005–2006 found that 41.6% in a sample of 4495 were vitamin D deficient. It was reported that vitamin D deficiency was significantly more common in those who were obese, self-reported poor health status, were hypertensive, had low levels of HDL cholesterol, and did not consume milk daily [1]. The increased incidence of insufficient or deficient vitamin D levels in adults is mostly due to the low levels of vitamin D found in food sources, less time spent outdoors, increased sunscreen use, increased use of protective clothing when outdoors, season, and latitude [2,3,4,5]. Vitamin D deficiency has been defined as having a 25-hydroxyvitamin D (25(OH)D) level <20 ng/mL, whereas vitamin D insufficiency is between 21–29 ng/mL, with a level of ≥30 ng/mL accepted as adequate by most experts [3,6].

As vitamin D aids in calcium absorption, one of its main effects is to enhance bone density; however, since vitamin D receptors have been located on multiple types of cells, it may be linked to increased chronic disease risk (cardiovascular disease, diabetes, dementia, etc.) and all-cause mortality [1]. It has been reported consistently in the literature that obese individuals have lower 25(OH)D levels compared to non-obese individuals. Wortsman et al. linked these lower levels to possible sequestration of synthesized vitamin D in the subcutaneous fat [7].

Vitamin D has also been associated with Type II diabetes mellitus (T2DM), however, many studies are epidemiological or cross-sectional in nature, with intervention studies rather scarce. Vitamin D receptors have been identified in pancreatic β-islet cells and may enhance insulin production and secretion [8]. Numerous epidemiological studies demonstrate an inverse relationship between 25(OH)D levels and several glycemic status measures, including fasting plasma glucose, oral glucose tolerance test (OGTT), and insulin resistance (HOMA-R) [9,10,11,12].

There is substantial evidence suggesting that low vitamin D levels may adversely affect cardiovascular health. 1,25 dihydroxy vitamin D (1,25(OH)_2_D) has been shown to regulate the major blood pressure regulating hormone, renin, in the kidneys, and thus acting as an inhibitor of the renin-angiotensin system, which is beneficial since an over-activation of the renin-angiotensin system can lead to hypertension [13,14]. Vitamin D has also been shown to be associated with triglyceride levels and high-density lipoprotein (HDL) levels [15,16]. Overall, vitamin D appears to have an association with all the factors that make up metabolic syndrome.

Finally, there seem to be several potential neuroprotective roles of vitamin D in relation to Alzheimer’s disease (AD). Active vitamin D (1,25(OH)_2_D) can bind to receptors in neuronal and glial cells throughout the essential cognitive regions of the brain (including the hippocampus, hypothalamus, and cortex) triggering neuronal protection via anti-inflammatory action. It also maintains nerve conduction integrity by promoting calcium homeostasis. AD is an inflammatory brain disease with microglia located near the β-amyloid plaques and increased release of tumor necrosis factor-alpha (TNF-α). Vitamin D can downregulate TNF-α production and promote cytokines and macrophages to increase β-amyloid clearance [17,18,19,20,21,22].

Thus, the aim of this study was to determine if deficient/insufficient vitamin D status is associated with risk factors (components of metabolic syndrome like blood pressure, fasting blood glucose, body fat, waist circumference, HDL, LDL and triglyceride levels; and cognitive dysfunction) of chronic diseases, such as cardiovascular diseases, diabetes, and dementia in 50 to 70 years old men and women. The age range of 50–70 years seemed appropriate for the study as the sample was old enough to have potential signs of metabolic syndrome and possible changes in cognition, but not so old that they were incapacitated to perform activities of daily living. 

## 2. Methods

### 2.1. Subject Characteristics

Eighty-seven recreationally active Caucasian adults, aged 50–70 years, were screened for this study. Recreationally active was defined as being physically active, but not participating in any structured exercise training program. Fifteen participants did not return for the study following the initial visit resulting in 72 subjects (54 females; 18 males) completing the study. Written informed consent was obtained from each participant prior to testing. The study was conducted in accordance with the Declaration of Helsinki and approved by the Institutional Review Board (IRB number: 1568) at the University of Oklahoma Health Sciences Center. Inclusion criteria were (1) males and postmenopausal females, aged 50–70 years old; (2) weight ≤300 lb (or ≤136.08 kg) based on DXA limits; (3) normal cognitive abilities as indicated by a score ≥27/30 points on the Mini Mental State Examination (MMSE); and (4) the ability to speak and understand English. Exclusion criteria were (1) premenopausal women; (2) individuals with artificial joints, or any other metal implants in the body; (3) individuals with physical disabilities, recent surgery, fractures and open wounds; (4) individuals with cognitive impairment that does not allow them to complete the study.

### 2.2. Study Design 

This study used a cross–sectional research design that examined the effect of vitamin D status on outcomes measures linked to chronic disease risk factors, such as excess body fat, waist circumference, A/G ratio, high blood glucose levels, high LDL and triglycerides, and low HDL levels. Men and women between the ages of 50 and 70 years were required to visit the campus on 3 separate occasions. The first visit required the subjects to sign a written informed consent, complete all questionnaires and to be familiarized with the computer-generated cognitive function assessment tests. The second visit required participants to visit the Health Center in order to have a fasting blood draw taken by trained professionals and during the third visit all other testing procedures were completed.

### 2.3. Procedures

#### 2.3.1. Questionnaires

Health Status Questionnaire: This is a screening questionnaire used to check for exclusion criteria or any other health issues that might impact the findings of the study. It has been prepared by referring medical history and health status questionnaires [23,24] for the healthy adult population typically assessed in our lab. Participants answered questions about past and current injuries, illnesses, medications, and current health status. 

Food intake: In order to assess the dietary and supplemental intake of vitamin D, each participant completed a vitamin D food frequency questionnaire (FFQ). This questionnaire was used to assess recalled intake of vitamin D over a predetermined period of time. The Calcium and Vitamin D Frequency Questionnaire developed by Taylor et al. 2009 [25] was used for this purpose, however, only the values specific to vitamin D were reported. The participant completed the questionnaire following which the portion size and frequency of consumption were reviewed with the participant. In order to prevent any reporting bias, the quantity of vitamin D present in each serving of the food item was not revealed to the participant. 

However, food frequency questionnaires can be prone to recall bias as they are based on food recall over a pre-determined time period. In contrast, a three-day dietary record can assess food intake over a short period of time and reduce the probability of recall bias [25]. Thus, in addition to the vitamin D FFQ, each participant also kept a detailed dietary record for any three usual non-consecutive days (two weekdays and one weekend day). The completed dietary record was then returned at the following visit and reviewed for completeness and portion sizes. The dietary record information was entered into FoodWorks nutrient analysis software [26] to obtain information regarding carbohydrate, protein, fat, as well as vitamin D intakes [27].

Sun Exposure Questionnaire: A sun exposure recall [28] recorded the average daily time spent outdoors and the amount of skin exposed to help assess sunlight exposure and illuminate the role of cutaneous vitamin D synthesis in vitamin D status.

Physical Activity Questionnaire: Physical activity levels over a 7-day period were assessed by the International Physical Activity Questionnaire (IPAQ) which asked questions about physical activity related to jobs, transportation, housework, recreation, and time spent sitting and classified individuals into 1 of 3 categories, low, moderate, or high [29,30].

Mental Status Questionnaire: The Mini Mental State Examination (MMSE) is a 30-point questionnaire that was used to measure cognitive impairment and screen for dementia. Participants were required to score ≥ 27/30 to indicate normal cognition and remain in the study [31,32].

#### 2.3.2. Descriptive Data

Resting brachial systolic and diastolic blood pressures (mmHg) were measured in a sitting position at the upper left arm using an automatic blood pressure measuring device (Omron IntelliSense Automatic Blood Pressure Monitor with Easy Wrap Cuff, model HEM-773AC, Vernon Hills, IL, USA). 

Height was measured without shoes, with the participant standing up tall to a nearest of 0.5 cm using a wall mounted stadiometer (Stadi-O-Meter, Novel Products Inc., Rockton, IL, USA). 

Weight was measured with minimal clothing, without shoes to a nearest of 0.1 kg using a digital weighing scale (TANITA BWB-800, TANITA, Japan). Body mass index (BMI) was calculated (kg/m^2^), and waist circumference was measured using a tape measure at the level of the navel.

#### 2.3.3. Pulse Wave Analysis (Central Blood Pressure and Arterial Stiffness)

Applanation tonometry (SphygmoCor, AtCor Medical, Sydney, Australia) and a high-fidelity strain-gauge transducer (Miller Instruments, Houston, TX, USA) were used to obtain blood pressure waveforms from the radial artery on the right arm [33]. Aortic blood pressure, arterial stiffness, and wave reflection were acquired from aortic blood pressure wave forms. Two measurements with an operator index >80 were procured, with the measurement having a higher operator index being used for further analysis. 

#### 2.3.4. Body Composition

A total body scan was performed using Dual Energy X-ray Absorptiometry (DXA, GE Lunar, Prodigy encore software version 10.50.086, Madison, WI, USA) to obtain total and regional measures of body fat and Android/Gynoid ratios (A/G ratio) [34]. A single technician performed all the scans and analyses to maintain accuracy and the lab % CV for body fat percentage was 1.87%.

#### 2.3.5. Blood Draws

A single venipuncture blood draw was obtained in the morning following an 8 h overnight fast performed by trained personnel. Each blood draw was divided into two 7.5 mL samples of blood. One blood sample was shipped to Diagnostic Laboratories of Oklahoma where the lipid panel (cholesterol, triglycerides, HDL, LDL (calculated), cholesterol/HDL Ratio (calculated)) and blood glucose levels were determined. The other blood sample was centrifuged to separate the serum and red blood cells, following which the serum was collected into microtubes and stored at −84 degrees Celsius. These microtubes were frozen until all the subjects completed the study and thawed only once to prevent protein degradation. The frozen serum samples were used to determine vitamin D levels using Immunochemiluminometric assay (ICMA) on the DiaSorin LIAISON instrument by LabCorp. Vitamin D sufficiency was defined as greater than or equal to 30 ng/mL. Normal expected coefficients of variation range from 3.7% to 7.3% [35]. The study was conducted for a duration of 1 year and 10 months and blood draws were taken throughout this time period.

#### 2.3.6. Cognitive Function

Cognitive function was assessed using the Automated Neuropsychological Assessment Metrics (ANAM) [36] test system consisting of a series of tests in a specific order, in cooperation with the Cognitive Science Research Center and the Center for the Study of Human Operator Performance (C-SHOP). The tests were implemented in the following order: (1) Simple Reaction Time (SRT): To determine the reaction time and vigilance, and visuo-motor response timing; (2) Code Substitution Learning (CDS): To assess complex scanning, visual tracking and attention; (3) Procedural Reaction Time (PRO): For measuring reaction time and coherence by following a simple set of mapping rules; (4) Mathematical Processing (MTH): Provides with an index of basic computational skills, concentration, and working memory; (5) Matching to Sample (M2S): Evaluates spatial processing and visuo-spatial working memory; (6) 2-Choice Reaction Time (CH2): This test uses a motor speed component to assess the processing speed and alternating attention of an individual; and (7) Code Substitution-Delayed (CDD): Examines the learning ability and visual recognition memory of an individual over an extended period of time.

### 2.4. Statistical Analyses

Data were analyzed using SPSS 24.0 (SPSS Inc., Chicago, IL, USA) software. All the dependent variables were tested for normality using the Shapiro-Wilk test. All the descriptive statistics are reported as median accompanied by minimum and maximum values. A one-way ANOVA was used to determine group differences between PA levels (3) for outcome measures with a normal distribution, while a Kruskal-Wallis test was used to examine group differences for outcome measures with distribution other than normal. Pearson correlation coefficients were used to assess the relationship between serum vitamin D levels and the risk factors of chronic diseases. Further, to examine group differences across the three levels of vitamin D status (deficient, insufficient, or sufficient) for the risk factors of chronic diseases and cognitive tests, a one-way ANOVA was used for variables with a normal distribution and Kruskal-Wallis test for variables with distribution other than normal. The association and sampling distribution between serum vitamin D status and PA levels, vitamin D status and cognitive function (ANAM scores), as well as, vitamin D status and vitamin D synthesis were determined using Chi-Square analysis. The level of significance for all analyses was set at *p* ≤ 0.05. For each outcome variable, the data were first analyzed as a group (n = 72) and then separated by PA levels (Low = 10, Moderate = 33, and High = 29).

## 3. Results

### 3.1. Demographics and Body Composition

Table 1 shows physical characteristics of participants based on physical activity levels. Percent body fat, BMI, and WC were significantly lower in participants with high level of PA compared to those with low PA levels (*p* < 0.05). 

### 3.2. Vitamin D Status, and Physical Activity Levels

Half of the subjects enrolled in this study (36/72) were classified as vitamin D deficient or insufficient with males (12/18 or 67%) constituting a greater proportion compared to females (24/54 or 44%). Additionally, a chi-square analysis involving vitamin D status and PA levels revealed that 70% of the participants in the low PA group (7/10), 52% (17/33) in the moderate PA group, and 41% (12/29) in the high PA group were vitamin D deficient and/or insufficient (*p* = 0.002). 

On average, the participants had normal lipid values with the exception of high LDL cholesterol (104.0 mg/dL (49.0–232.0 mg/dL)) which remained elevated regardless of PA levels. Although non-significant, participants with higher PA levels had higher HDL cholesterol and lower triglyceride levels. Participants in moderate and high PA groups had significantly higher vitamin D levels in comparison to the low PA group (*p* < 0.05) (Table 2).

### 3.3. Total Caloric and Macronutrient Intakes

Table 3 presents the three-day average total caloric intakes and macronutrient percentages of the total intake. Subjects met the acceptable macronutrient distribution range for carbohydrates: 45–65% of total kcals (48.61%), proteins: 10–35% of total kcals (16.95%), and fats: 20–35% of total kcals (33.76%). 

The total kcals consumed were significantly higher for the high (*p* = 0.001) and moderate PA group (*p* = 0.003) compared to the low PA group.

### 3.4. Central Hemodynamics

There were no significant statistical differences between PA levels for brachial systolic or diastolic blood pressures and heart rate measures, with all blood pressures (SBP/DBP) in the normal range of 117–127/75–82 mmHg and all heart rates in the normal range of 61–74 beats per minute. In general, there were also no significant differences between PA levels for measures of central blood pressures or arterial stiffness. 

### 3.5. Dietary and Supplemental Vitamin D Intake

There were no significant differences between PA levels for vitamin D intakes or vitamin D supplementation (Table 4). There was a low and non-significant correlation (*r* = 0.17) between dietary vitamin D intake and circulating levels of vitamin D with minimal influence of PA levels (low PA: *r* = 0.30; moderate PA: *r* = 0.05; and high PA: *r* = 0.17) on this relationship.

Considering the scarcity of vitamin D food sources, average dietary vitamin D (89 IU for this study population vs. RDA of 600 IU for individuals aged 51–70 years) accounted for only about 3% of the circulating (25(OH)D) vitamin D levels. However, a significant moderate positive correlation (*r* = 0.54; *p* = 0.01) was observed between supplemental intake of vitamin D and its circulating levels, thus explaining approximately 30% of the variance in the circulating vitamin D levels.

### 3.6. Sun Exposure

Individuals with high and moderate PA levels had significantly higher sun exposure scores than those in the low PA group (22 (3–46), 18 (2–44) vs. 10 (0–18); *p* = 0.02). Skin exposure scores were significantly higher for individuals with moderate PA levels compared to low PA levels (14 (7–22) vs. 9 (7–14); *p* = 0.04). There were no significant correlations between serum vitamin D levels and the various components of the sun exposure questionnaire.

### 3.7. Cognitive Function

Prior to statistical analyses, a detailed examination of the cognitive data was conducted to ensure the validity of the data and to screen for outliers. There were no significant group differences across the three levels of vitamin D status (deficient, insufficient, and sufficient) for any of the cognitive test items and no significant correlations existed between the cognitive test items and serum vitamin D levels (Table 5).

### 3.8. Serum Vitamin D and Chronic Risk Factors

When analyzing the entire sample, circulating levels of vitamin D were negatively related to the risk factors of chronic diseases, including glucose (*r* = −0.38; *p* = 0.01), triglycerides (*r* = −0.27; *p* = 0.02), and A/G Ratio (*r* = −0.32; *p* = 0.01).

When the entire sample was considered, there was a significant difference across the three levels of vitamin D status (deficient, insufficient, and sufficient) for each risk factor of chronic disease, including glucose levels (*p* = 0.001), triglyceride levels (*p* = 0.03), percent body fat (*p* = 0.04), and A/G Ratio (*p* = 0.007), where each of these variables decreased incrementally as vitamin D status improved across the three levels. 

### 3.9. Serum Vitamin D Levels/Vitamin D Status

There was a significant (*p* = 0.04) seasonal influence on sunlight exposure associated with the date that the participant had their vitamin D status measured with their actual vitamin D status. Five of the 16 (31%) subjects tested between November-February were vitamin D deficient vs. only 4/56 (7%) tested during March-October. Also, 30/56 (54%) tested between March-October were vitamin D sufficient vs. only 6/16 (38%) tested between November-February. Finally, 30/36 (83%) of the participants with sufficient (≥30 ng/mL) vitamin D status were tested between March to October vs. only 6/36 (16.7%) of those tested between November to February.

## 4. Discussion

The unique findings of this study include the fact that half of the participants were classified as being vitamin D deficient or insufficient; that vitamin D status was inversely related to several risk factors of chronic diseases; and that higher levels of physical activity were associated with sufficient vitamin D levels and lower values for several chronic disease risk factors.

Cutaneous synthesis of vitamin D via sun exposure, vitamin D supplementation, and dietary intake of vitamin D are the primary contributors to vitamin D status. However, low levels of vitamin D in food sources, along with decreased levels of physical activity in middle aged to older adults, often results in reduced sun exposure. The reduction in sun exposure can lead to the limited cutaneous synthesis of vitamin D which increases the susceptibility of vitamin D insufficiency/deficiency and predisposes individuals to risk factors of chronic diseases associated with vitamin D deficiency [37]. Thus, the age range of 50 to 70 year old seemed very appropriate for the current study, since 50% of the study population was classified as vitamin D deficient/insufficient (<30 ng/mL).

The participants were classified as overweight/obese, due to elevated BMI (25.75 kg/m^2^ (19.4–50.2 kg/m^2^)) and percent body fat (37.6% (17.2–56.5%)). BMI, percent body fat and waist circumference were inversely related to physical activity levels, with the participants in the low PA group having the highest BMI, percent body fat, A/G ratio, and waist circumference, as well as having the lowest serum vitamin D levels. These results are consistent with previous literature reporting an association between inadequate circulating vitamin D levels and obesity in similar age ranges [7,11,38,39,40]. Some authors hypothesize that the increased amount of adipose tissue present in obese individuals might sequester vitamin D since it is fat soluble [7]. Nevertheless, others postulate that the lower vitamin D levels found in obese individuals are related to volumetric dilution. As the volume of fat increases with obesity, a greater amount of vitamin D is required to saturate it and to attain serum vitamin D levels similar to those of normal weight individuals [41]. Although there is inconclusive evidence to establish the direction of causality between vitamin D and fat, most studies fail to demonstrate vitamin D deficiency as the cause for the development of obesity [42]. Therefore, the notion that increased body fat decreases vitamin D levels through sequestration and/or dilution at least in part helps to explain vitamin D deficiency/insufficiency in overweight/obese individuals. Thus, the increased body fat in the current study sample, particularly in individuals with low PA levels, can restrict vitamin D synthesis both by sequestration/volumetric dilution and lack of exposure to sunlight. 

Due to the negative relationship between vitamin D and fat, numerous studies emphasizing the importance of vitamin D intake based on body size/weight have formed an effective strategy for coping with vitamin D insufficiency/deficiency in overweight/obese individuals [4,43,44,45,46]. These studies suggest that increasing outdoor PA will promote vitamin D formation, both by weight loss and cutaneous synthesis of vitamin D through sensible sun exposure. Consistent with these studies, the present study found that an increase in PA was associated with decreased frequency of vitamin D insufficiency/deficiency and lower BMI, percent body fat and waist circumference. 

Since adipose tissue is a highly metabolic tissue that is involved in lipid and glucose metabolism, the high incidence of overweight/obese subjects in the current study may place these individuals at risk for being in a state of chronic inflammation with adipose dysfunction, potentially linked to increases in atherosclerosis [47,48,49]. The subjects in this study had elevated LDL levels, which, once oxidized, initiates a cascade leading to inflammation, macrophage infiltration, and endothelial cell adhesion that can also lead to atherosclerosis.

Although statistically non-significant, higher PA levels were associated with increased HDL levels and decreased triglycerides, a cardiovascular risk factor associated with low vitamin D status [15]. Also, higher vitamin D levels were accompanied by lower average blood glucose levels as the PA levels of the individuals improved from low towards high (unreported data). Vitamin D enhances the synthesis of insulin from the beta-cells of the pancreas and also accelerates the conversion of proinsulin to insulin [11]. Additionally, the presence of vitamin D receptors in the beta-cells further support the idea that vitamin D regulates insulin secretion at least partly, and its deficiency/insufficiency can result in decreased insulin sensitivity at the target tissues, thereby causing or worsening diabetes. As physical activity enhances insulin sensitivity, increase in physical activity can prevent diabetes both by decreasing insulin resistance and by increasing cutaneous vitamin D synthesis [50]. 

When considering body composition, glucose, and lipid panel values of the subjects, it becomes apparent that improved dietary practices could improve these outcome variables and decrease risk factors of chronic diseases [50]. Although the participants met the acceptable macronutrient distribution range (AMDR), the percent of kcals from fat was at the upper end of this range while fiber intake was lower for males and borderline for females (unreported data). A decrease in fat intake and an increase in fiber intake would have a positive effect on body fat and lipid levels, since dietary fiber decreases cholesterol levels in the body [51].

Peripheral and central blood pressures were normal for participants in this study. Central pressure is better related to future cardiovascular diseases, since the heart, kidneys and major arterial supply of the brain is controlled by aortic pressure [52]. The large number of overweight/obese subjects in the study may be at increased risk for hypertension, with 25% of the subjects taking some form of hypertension medication. Individuals with sufficient vitamin D levels may exhibit better blood pressure control, since vitamin D regulates renin in the kidneys, the major blood pressure regulating hormone [53]. 

No significant differences were found between vitamin D status for tests of executive function (attention, processing speed, working memory, spatial processing) in this study. Contrary to this, Annweiler et al. [54] found that lower circulating vitamin D levels predicted executive dysfunction. Annweiler et al. [55] also concluded that <10 ng/mL seems to be the threshold that is most associated with cognition, as this would signify chronic hypovitaminosis D and has probably contributed to brain dysfunction over an extended period of time. Obesity, elevated LDL levels, and high dietary fat intake may expose the current study population to risk of cognitive dysfunction later in life. Although vitamin D status by itself is not enough to diagnose cognitive dysfunction, it is part of the equation, and with many biological targets throughout the body, vitamin D levels most likely contribute to a wide variety of symptoms seen in older adults with cognitive disorders.

### Limitations

This study has certain limitations. Due to the cross-sectional study design, this data is limited in drawing a causal relationship between vitamin D status and the risk factors of chronic diseases. Additionally, the use of blood pressure or cholesterol medication was not controlled. Sixteen subjects had their vitamin D status tested during the winter when the ability of cutaneous formation of vitamin D is negligible. Also, the sun exposure questionnaire did not record any information regarding the use of sunscreen or the type of clothing worn. Furthermore, there are potential limitations associated with multi-testing and multi-factorial effects which the authors tried to control by minimizing the learning effects related to cognitive testing. All the subjects underwent only one familiarization session to understand what was expected during the actual test. Moreover, ANAM is a standardized test battery given in a predetermined manner, with each of the tests measuring different aspects of cognitive function with minimal overlap. Finally, the limited sample size (n = 72) and gender discrepancy (54 females, 18 males) may affect variances and ANOVA results, increasing the chances of Type II error, or a failure to detect any significant effect which may otherwise be present.

## 5. Conclusions

In conclusion, deficient/insufficient vitamin D levels are linked to the risk factors (glucose, triglycerides, percent body fat, and A/G ratio) of chronic diseases in men and women aged 50 to 70 years. Additionally, individuals with higher PA levels had greater serum vitamin D concentration, and lower BMI, WC, and percent body fat in comparison to those with low PA levels, emphasizing the importance of PA for maintenance of vitamin D levels either directly through cutaneous vitamin D synthesis, or indirectly through reduction of body fat. Although only intervention studies can consistently draw a conclusion on the role of vitamin D for the prevention of chronic diseases, the results of this study suggest that maintaining adequate vitamin D levels and including vitamin D assessments as a part of routine physical examination is essential especially in the elderly, obese and sedentary individuals, those living at higher latitudes, and individuals with a darker skin color. 

## Figures and Tables

**Table 1:** Physical characteristics based on physical activity (PA) levels.

Variables		Groups			*p*
	Total	Low PA (*n* = 10)	Moderate PA (*n* = 33)	High PA (*n* = 29)	
Age	60.9 (50–70.8)	61.6 (51.8–66.0)	60.9 (50.6–69.3)	60.9 (50.9–70.8)	0.88
Height	165.0 (146.0–191.0)	164.0 (146.0–170.5)	165.0 (151.0–184.5)	162.5 (150.0–191.0)	0.18
Weight	76.25 (49.5–126.9)	84.4 (49.5–146.5)	77.7 (55.7–123.6)	67.1 (49.9–97.5)	0.13
BMI	25.75 (19.4–50.2)	30.6 (21.6–50.2)	26.0 (20.5–40.7)	25.3 (19.4–31.6)	0.03 ^δ^
WC	37.75 (28.5–57.0)	42.0 (32.5–57.0)	38.5 (31.5–50.0)	37.0 (28.5–44.0)	0.03 ^δ^
%BF ^¶^	37.6 (17.2–56.5)	47.2 (34.1–56.5)	39.4 (17.2–55.5)	33.9 (18.2–45.8)	0.001 *
A/G Ratio ^¶^	1.01 (0.64–1.50)	0.97 (0.73–1.02)	1.02 (0.65–1.39)	1.04 (0.64–1.50)	0.64

Data presented as median (minimum-maximum value); PA, physical activity; BMI, body mass index; WC, waist circumference; %BF, percent body fat; A/G Ratio, android/gynoid ratio; *p*, *p*-value of one-way ANOVA or Kruskal-Wallis test. ^¶^ normal distribution; * high PA vs. low PA; ^δ^ high PA vs. moderate and low PA.

**Table 2:** Participant blood values at different PA levels.

Variables	Total(*n* = 72)	Groups			*p*
		Low PA (*n* = 10)	Moderate PA (*n* = 33)	High PA (*n* = 29)	
T Chol	190.5 (127–318)	192 (153–231)	188 (127–267)	191 (158–318)	0.90
TG	103 (42–835)	94 (43–252)	120 (51–272)	85 (42–219)	0.07
HDL ^¶^	63 (29–113)	63 (37–98)	59 (38–100)	69 (40–113)	0.07
LDL	104 (49–232)	101 (74–144)	105 (49–177)	108 (51–232)	0.66
GLU	90 (75–307)	90 (78–139)	88 (81–130)	91 (75–152)	0.61
VIT D	29.8 (7.6–57.7)	20.8 (10.1–36.4)	29.3 (17.8–57.7)	32.4 (7.6–53.1)	0.04 ^#^

Data presented as median (minimum-maximum value); T Chol, total cholesterol; TG, triglycerides; HDL, high-density lipoproteins; LDL, low-density lipoproteins; GLU, glucose; VIT D, vitamin D; *p*, *p*-value of one-way ANOVA or Kruskal -Wallis test. ^¶^ normal distribution; ^#^ high and moderate PA vs. low PA.

**Table 3:** Three-day average total caloric intake and percentages of total caloric intake at different physical activity levels.

Variables	Total(*n* = 72)	Groups			*p*
		Low PA (*n* = 10)	Moderate PA (*n* = 33)	High PA (*n* = 29)	
Kcals	1747.5 (667.6–3628)	1232 (970.5–2063)	1759 (667.6–3133)	1832 (1115–3628)	0.004 ^#^
% Kcal Fat ^¶^	33.76 (14.23–56.96)	34.88 (20.81–47.72)	33.10 (14.23–56.96)	33.98 (14.93–50.54)	0.65
% Kcal PRO ^¶^	16.95 (6.10–29.78)	17.38 (11.15–25.39)	17.01 (6.10–29.78)	16.63 (10.37–28.59)	0.95
% Kcal CHO ^¶^	48.61 (23.83–74.04)	43.88 (26.98–54.05)	49.24 (23.83–65.67)	47.17 (33.56–74.04)	0.62

Data presented as median (minimum-maximum value); Kcals, calories; % Kcals, % of daily calories from nutrient; *p*, *p*-value of one-way ANOVA or Kruskal-Wallis test. ^¶^ normal distribution; ^#^ high and moderate PA vs. low PA.

**Table 4:** Dietary and supplemental vitamin D.

Variables	Total (*n* = 72)	Groups			*p*
		Low PA (*n* = 10)	Moderate PA (*n* = 33)	High PA (*n* = 29)	
Vit D (IU)	38.44 (0.0–616)	17.88 (3–85.76)	45.28 (0.0–321.24)	38.48 (0.0–616)	0.56
Vit D (Supp) ^¶^	450 (0.0–10,000)	400 (0–10,000)	700 (0–6500)	600 (0–3500)	0.69

Data presented as median (minimum-maximum value); Vit D IU, dietary vitamin D in international units; Vit D Supp, supplemental vitamin D in international units; *p*, *p*-value of one-way ANOVA or Kruskal-Wallis test. ^¶^ normal distribution.

**Table 5:** Throughput scores.

	Total (25(OH)D)	Group 1 (Deficient)	Group 2 (Insufficient)	Group 3 (Sufficient)	*p*	*r* ^a^
*n*	72	9	27	36		
ANAM Tests						
Simple Reaction Time	216.53 (54.38–275.33)	194.66 (54.38–275.33)	215.61 (159.82–268.88)	226.48 (161.41–262.01)	0.119	0.07
Code Sub Learning ^¶^	37.14 (19.13–58.69)	36.10 (25.32–48.18)	36.83 (25.53–52.80)	37.90 (19.13–58.69)	0.860	−0.14
Proced React Time ^¶^	97.17 (62.79–124.39)	87.89 (72.39–124.39)	97.97 (62.79–115.95)	96.36 (69.73–116.03)	0.993	0.03
Math Processing ^¶^	25.33 (9.17–41.04)	26.19 (13.51–41.04)	25.94 (13.09–34.78)	24.79 (9.17–39.71)	0.837	0.02
Match to Sample ^¶^	28.31 (8.95–50.16)	23.98 (14.31–41.55)	28.91 (17.93–50.16)	27.12 (8.95–40.93)	0.203	−0.10
2-Choice React Time	125.19 (2.91–161.22)	121.24 (105.35–149.94)	123.93 (73.39–150.65)	126.54 (2.91–161.22)	0.937	−0.10
Code Sub Delayed ^¶^	32.57 (12.54–60.72)	29.63 (23.97–39.28)	40.94 (12.54–60.72)	32.14 (14.82–57.15)	0.271	0.05

Data presented as median (minimum-maximum value). ^a^ Pearson correlation between vitamin D level and Automated Neuropsychological Assessment Metrics (ANAM) Throughput score. Throughput is a hybrid measure of reaction time and accuracy. Reported as correct responses per minute. Higher values indicate better performance. *p*; *p*-vale of one-way ANOVA or Kruskal-Wallis test: Throughput scores across levels of vitamin D status (Deficient, Insufficient, Sufficient). ^¶^ normal distribution.
